# The reliability of using gingival crevicular blood to measure blood glucose and hba1c levels in the dental setting: a systematic review and meta-analysis

**DOI:** 10.1007/s00784-024-05685-4

**Published:** 2024-05-04

**Authors:** Omid Fakheran, Bulcsu Bencze, Irene Mischak, Daniel Vegh, Michael Payer

**Affiliations:** 1 Division of Oral Surgery and Orthodontics, Department of Dentistry and Oral Health, Medical University of Graz, Billrothgasse 4, Graz, 8010 Austria; 2 Department of Prosthodontics, Semmelweis University, Szentkiralyi utca 47, Budapest, 1088 Hungary

**Keywords:** Bleeding on probing, Diabetes mellitus, Gingival crevicular blood, Screening

## Abstract

**Objective:**

There are 500 million patients living with diabetes mellitus worldwide and 50% of them remain undiagnosed. Routine periodontal probing provides gingival crevicular blood in patients with gingivitis. Gingival blood may be useful for diabetes screening without the need for any expensive, painful or time-consuming method by using convenient glucometers. Therefore, the objective of this systematic review and meta-analysis is to answer the question to “is there a difference in glucose or HbA1c levels (O) in patients with positive gingival bleeding (P) measured on gingival crevicular blood (GCB) (I) compared to finger prick capillary blood (CB) (C).

**Materials and methods:**

The authors performed an electronic search of six databases using identical MeSH phrases. Only human clinical studies without limitations on the year of publication were considered. Data extraction was done by using standardized data collection sheets. Risk of bias assessment were conducted using QUADAS-2 and QUADAS-C. Meta-analyses were carried out with the random effects model to aggregate the correlation coefficients and the difference between the means between gingival and capillary blood reading, using 95% confidence intervals.

**Results:**

The database and manual search yielded 268 articles, from which the selection procedure provided 36 articles for full-text screening, and the final pool of eligible articles composed of 23 studies with 1680 patients. Meta-analysis results on glycemic levels showed differences between the GCB and CB procedures in patients with and without diabetes with values of -6.80 [-17.35; 3.76] and − 4.36 [-9.89; 1.18], respectively. Statistically significant correlations were found (*p* = 0.001) between GCB and CB measurements in patients with (0.97 [0.927; 0.987]) and without diabetes (0.927 [0.873; 0.958]).

**Conclusion:**

Gingival blood could prove to be useful to identify patients with undiagnosed diabetes when the necessary amount of uncontaminated blood is present. However, this technique is limited by the possibility of contamination, prandial status and inaccuracies, so it is unsuited to address the patient’s glycemic control accurately.

## Introduction

Diabetes mellitus (DM) is known to be one of the major global epidemic diseases with more than 500 million patients worldwide of which 50% are undiagnosed cases. It is significantly associated with mortality and morbidity, conferring a substantial burden to the healthcare system with an approximate of USD 960 billion dollars in expenditure [1, 2]. DM is a chronic metabolic disorder that leads to hyperglycemia, which raises multiple complications caused by micro- and macroangiopathy [3]. Chronic hyperglycemia leads to increased pro-inflammatory cytokine levels both systematically and locally which leads to increased occurrence of periodontitis, significant risk of tooth loss, delayed wound healing and impaired response to infections [4, 5]. Moreover, poorly controlled DM increases the risk and severity of periodontitis, peri-implantitis, and diminishes the effectivity of periodontal treatment therapy [6]. However, there is still no evidence that dental implant surgery is contraindicated in patients with prediabetes or well-controlled DM [7]. Therefore, screening patients for undiagnosed DM, and also checking the quality of glycemic control is an important aspect for dental surgeries.

There are four ways of diagnosing DM according to the American Diabetes Association, HbA1c level, fasting plasma glucose (FPG), oral glucose tolerance test (OGTT) and random plasma glucose test (RPG). OGTT is impractical at the average dental setting, while FPG is only suggested when non-invasive procedures are planned, for eight hours of fasting are required before measuring. For HbA1c, values higher than 6.5% (48 mmol/mol) indicate DM, in case of FPG measurement the value is 6.9 mmol/L (125 mg/dl), whereas for RPG only severe DM can be detected with values higher than 11.1 mmol/L (200 mg/dl) [8].

The U.S. Preventive Services Task Force determined there is sufficient evidence that lifestyle interventions can prevent or delay progression to type II DM [9]. Moreover, early diagnosis of DM is key to avoid the microvascular consequences of the disease since approximately 25% of newly diagnosed patients have already developed at least one complication [10]. Appropriate screening devices and standardized methods are crucial to prevent this potentially inauspicious life condition. Dental teams can assist in the early detection, diagnosis and treatment of DM and, secondarily, other chronic conditions, such as cardiovascular disease [11].

Routine periodontal probing produces gingival crevicular blood (GCB) in patients with gingivitis or periodontitis. In recent years, some published clinical studies showed that the GCB may be useful for DM screening without the need for any extra and uncomfortable procedure like the need for finger puncture with sharp lancets. Currently, the glucometer is the conventional device employed for capillary finger-stick blood glucose level determination which can be also used to measure the glucose content of gingival blood [12–14]. Routine probing during a periodontal examination is more familiar to the practitioners and less traumatic for the patients. Plasma HbA1c levels represent the last two to three months of average systemic blood glucose levels, which gives additional insight on glycemic control besides direct blood glucose measurements [15]. Even in the cases of low gingival crevicular bleeding, a glucose measurement is possible with the help of the self-monitoring device. In addition, the sampling procedure is much easier to perform and less time-consuming [16].

To the best of our knowledge, there are no systematic reviews published in the literature in this regard. Hence, the aim of this study is to assess the reliability of using gingival crevicular blood for identifying patients with undiagnosed DM and assessing their quality of glycemic control in the dental setting. Moreover, we aim to interpret the probable variations in the results obtained by the researchers who have examined the feasibility and acceptability of using gingival crevicular blood as an alternative to capillary blood (CB) to measure blood glucose and HbA1c levels.

## Material & methods

### Search strategy

**Fig. 1:**
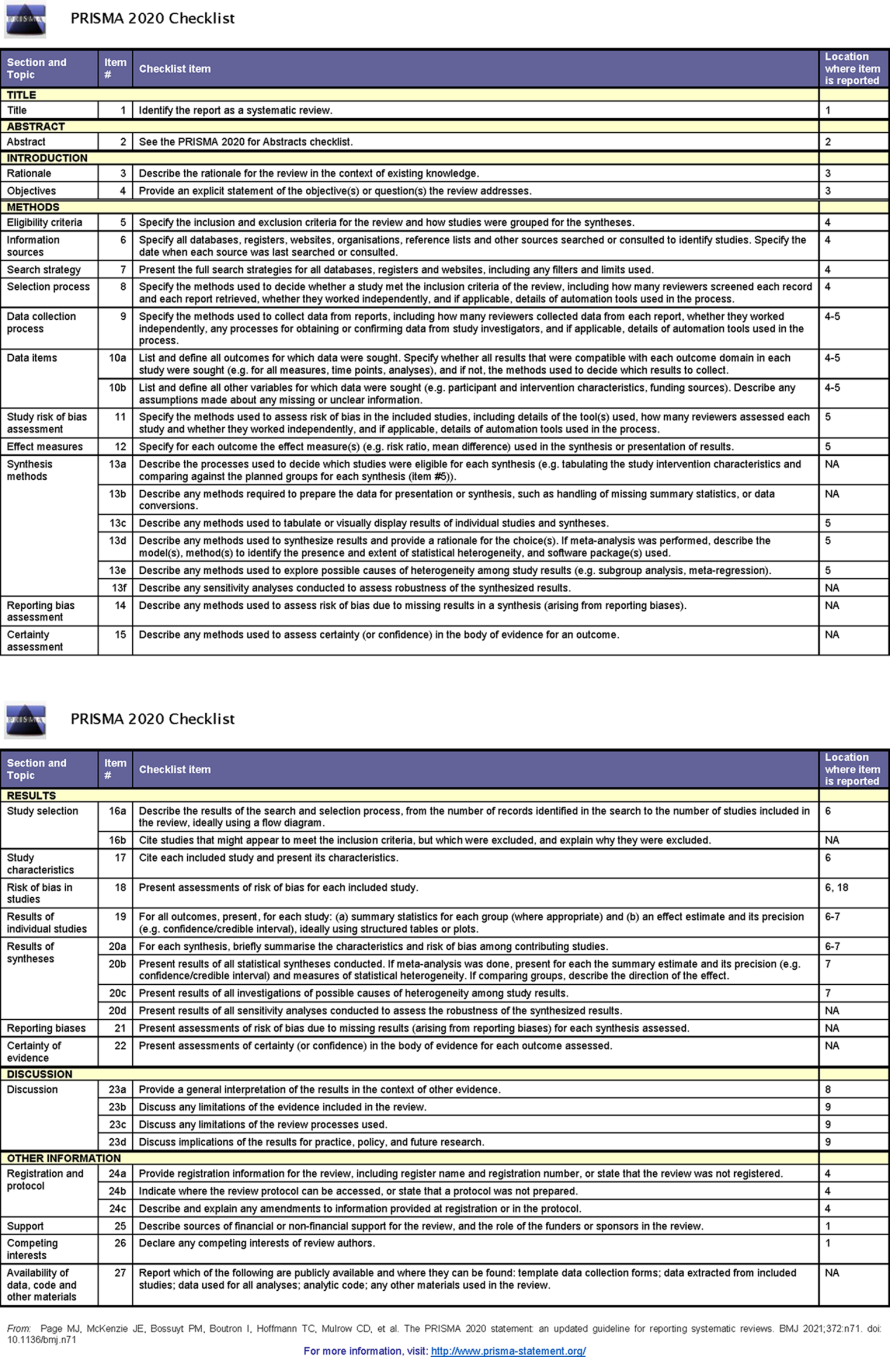
PRISMA checklist 2020

This systematic review was performed according to the Cochrane Handbook and reported according to the Preferred Reporting Items for Systematic Reviews and Meta-analyses (PRISMA) statement items (Fig. 1.) [17]. We also developed and submit a protocol to the International Prospective Register of Systematic Reviews (PROSPERO) database (ID: CRD42022372748).

We applied the literature search to answer the following focused question: is gingival crevicular blood a reliable tool to measure HbA1c and glucose levels in the dental setting?

In this regard, the following PICO framework was used:

- Participants (P) are the adult individuals with positive bleeding on probing.
- The intervention (I) is collecting the gingival crevicular blood and using a glucometer to estimate the blood glucose level.
- The comparison (C) is using other sources of blood to measure the blood glucose level.
- The Outcome (O) is measuring Hemoglobin A1c and glucose level.

### Data sources & search strategy

We performed an electronic literature search using a wide range of computerized databases, including MEDLINE, Cochrane Library, PsycINFO, Web of Science, Google Scholar and Scopus on 15 July 2023. We did not use any filters based on language or publication date in our electronic literature search. We used the following search terms and protocols in this systematic review:

((gingival crevicular blood) OR (crevicular blood)) AND ((((((((Diabetes mellitus) OR (Hyperglycemia)) OR (High blood glucose levels)) OR (type 2 diabetes)) OR (Finger stick blood)) OR (Finger prick blood)) OR (Glucometer)) OR (glucose)) OR (hemoglobin A1c)).

The terms and keywords were adapted for each database as necessary. We also performed an extensive manual search encompassing the bibliographies and citations of the included papers and review articles. Furthermore, we searched the websites that list ongoing clinical trials: *(*http://clinicaltrials.gov, http://www.centerwatch.com/http://www.clinicalconnection.com*).*

### Eligibility criteria

The inclusion criteria for this study were as follows: (1) Human clinical studies comprising randomized controlled trials, prospective studies, retrospective studies, and case series; (2) The investigations involved collecting the gingival crevicular blood and at least one other blood sample to measure hemoglobin A1c and/or glucose levels; (4) a minimum of twenty patients; (6) no deadline for publication date.

The exclusion criteria were as follows: (1) nonclinical and animal studies, case reports, review articles, and commentaries (2) the unavailability of full-text articles; and (5) full-text papers written in a language other than English.

### Study selection & data extraction

At the first study selection step, two reviewers (OF & MP) independently screened the (1) titles and (2) abstracts. Subsequently, the full text of all eligible studies was obtained and checked by the same reviewers [17]. Disagreements were resolved through discussion. After that, we excluded the publications that did not meet the eligibility criteria and we recorded the reasons for exclusion.

Afterwards we extracted and assimilated data on a piloted, standardized data collection sheet. We classified all the data in relation to year of publication, country, measurement methods, patient characteristics, confounding factors and outcomes according to the aims of this study.

### Quality assessment

Two reviewers (OF & MP) independently conducted a risk of bias assessment using QUADAS-2 (Quality Assessment of Diagnostic Accuracy Studies-2) and QUADAS-C (Quality Assessment of Diagnostic Accuracy Studies–Comparative instruments [18, 19].

The QUADAS-C tool can assess risk of bias in test comparisons undertaken in comparative accuracy studies. QUADAS-C is an extension of QUADAS-2 [18]. The QUADAS tool was used to assess risk of bias and concerns regarding patient selection, index test, reference standard, and flow and timing, with each domain being classified into one of three categories: (i) high risk of bias; (ii) unclear risk of bias; and (iii) low risk of bias. T [19, 20]. Any discrepancy between reviewers in quality ratings was resolved by discussion and consensus.

In addition, we assigned a level of evidence for each article using the classification system described by Wright et al. [21].

### Statistical analysis

The meta-analysis was conducted using R software (version 4.0.2, R Foundation for Statistical Computing, Vienna, Austria) to pool correlation coefficients and to calculate the mean differences (MD) [22]. The analysis was conducted by using the random effects model assuming significant between-study heterogeneity. To calculate the difference between the means, continuous variables, and 95% confidence intervals were used. To calculate the study MDs and pooled MD, the sample size, the mean and the corresponding standard deviation (SD) was extracted from each study (in each group separately). We reported the results as the experimental group minus the control group values. Subsequently, the Pearson correlation coefficients from the included studies were pooled and analyzed, forest plots were created for both analyses [23]. This was carried out once for patients with DM and once for patients without DM independently. Heterogeneity across the studies was assessed using the I^2^ test [24]. An I^2^ value greater than 75% was considered high.

## Results

### Study selection

**Fig. 2:**
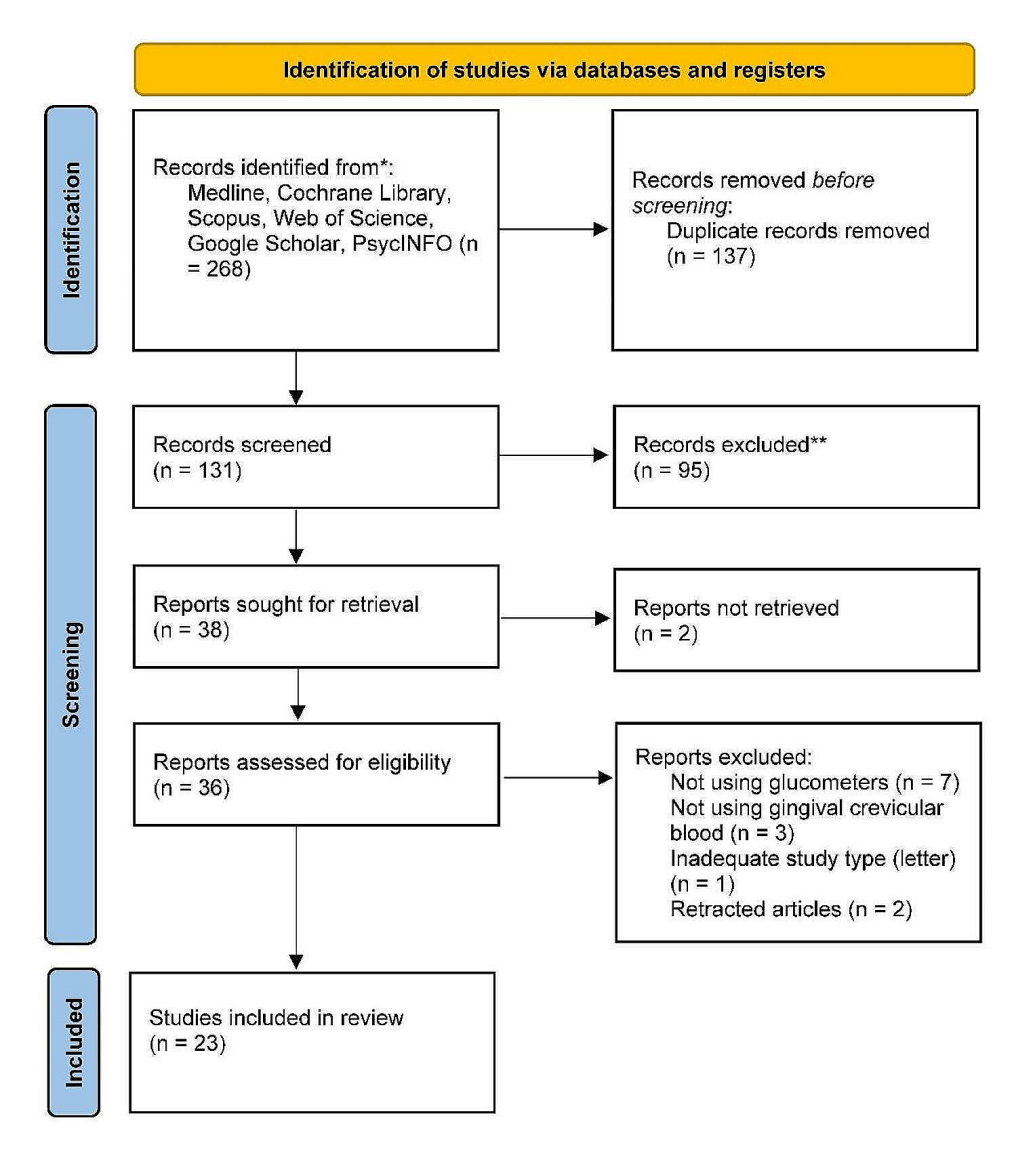
Prisma flowchart 2020

A total of 268 possibly relevant articles were identified through the search strategy. After completing the screening titles and eliminating duplicates, 131 studies were retrieved, and their abstract versions were collected for further assessment (Kappa value = 0.87). We selected Thirty-six studies based on the abstract screening phase. A manual search for the reference lists of the 36 studies revealed no additional qualifying paper. Then we assessed the full text version of these 36 studies. According to the results of the full article review stage, thirteen articles were excluded. The reasons for excluding full-text articles are presented in Fig. 2. PRISMA flow chart of selection process.

Finally, we included 23 eligible clinical studies to this systematic review and meta-analysis study according to predefined inclusion and exclusion criteria.

### Study characteristics

The included articles were published between 1993 and 2023. The majority of the literature is from India with 13 studies [13, 25–35], two from Kuwait [36, 37] and the USA [38, 39], and one from China [14], Italy [12], Pakistan [40], Jordan [41], Germany [42] and Iran [43] respectively. All in all, the studies included 1680 mostly middle-aged patients with an average age of 44.4 and with a roughly equal distribution of sex with slightly more females (51%). The basic characteristics of the included studies can be found in Table 1.

**Table 1:** Basic characteristics of the included studies

First Author	Year of publication	Country	Number of patients	Percentage of females	Results of GCB and CB readings	Conclusion
Robert C. Parker	1993	USA	50	66	Not reported	GCB can provide an acceptable source for measuring blood glucose in the study’s specific glucose self-monitor.
T. Beikler	2002	Germany	45	53,3	Not reported	GCB collected during diagnostic periodontal examination may be an excellent source of blood for glucometer analysis.
H-P Müller	2004	Kuwait	46	56,52	GCB: 4.33 ± 2.11 mmol/L CB: 5.72 ± 2.06 mmol/L	The present study failed to provide any evidence for the usefulness of GCB for testing.
Hans-Peter Müller	2005	Kuwait	46	NA	GCB: min 21 mg/dL max 180 mg/dL CB: min 25 mg/dL max 207 mg/dL	Screening for elevated blood glucose levels should not be performed in GCB oozing from the sulcus after routine periodontal probing.
Yousef Saleh Khader	2006	Jordan	60	NA	GCB: 125.4 ± 60.7 mg/dL CB: 131.9 ± 61.1 mg/dL	GCB can provide an acceptable source for measuring blood glucose level.
Shiela M. Strauss	2009	USA	46	65	GCB: min 68 mg/dL max 234 mg/dL CB: min 71 mg/dL max 203 mg/dL	With minimal cost and a limited investment of time for patients and clinicians, dental professionals can play a critical role in supporting their patients’ overall health.
Mohammad Reza Talebi Ardakani	2009	Iran	60	50	DM GCB: 240.27 ± 74.95 mg/dL non-DM GCB: 97.03 ± 31.67 mg/dL DM CB: 269.73 ± 84.91 mg/dL non-DM CB: 111.4 ± 36.35 mg/dL	There is a high correlation between GCB and CBLs among patients with DM and healthy subjects, regardless of gender.
Subodh Gaikwad	2013	India	30	NA	GCB: 96.48 ± 62.38 mg/dL CB: 131.36 ± 87.06 mg/dL	GCB may serve as a potential source for screening of blood glucose during routine periodontal examination.
Harmanpreet Kaur	2013	India	50	34	DM GCB: 230.1 ± 99.4 mg/dL non-DM GCB: 105.4 ± 25.9 mg/dL DM CB: 256.2 ± 111 mg/dL non-DM CB: 122.5 ± 27.7 mg/dL	GCB collected during diagnostic periodontal examination may be an excellent source of blood for glucometric analysis.
Neema Shetty	2013	India	100	43	DM GCB: 193.52 ± 74.93 mg/dL non-DM GCB: 97.2 ± 15.7 mg/dL DM CB: 218.54 ± 84.04 mg/dL non-DM CB: 104.48 ± 13.84 mg/dL	GCB collected during diagnostic periodontal examination may be an excellent source of blood for glucometric analysis.
Shivani Dwivedi	2014	India	75	42,66	GCB: 101.46 ± 24.31 mg/dL CB: 108.4 ± 27.86 mg/dL	GCB collected during diagnostic periodontal examination may be an excellent source of blood for glucometric analysis.
Amit Gupta	2014	India	30	45,8	DM GCB: 172.27 ± 5.02 mg/dL non-DM GCB: 109.8 ± 5.11 mg/dL DM CB: 167.8 ± 8.87 mg/dL non-DM CB: 106.93 ± 1.8 mg/dL	GCB is a reliable and definitive indicator for analysis of glycemic status of an individual.
Puja Debnath	2015	India	50	38	DM GCB: 210.56 ± 17.26 mg/dL non-DM GCB: 118.76 ± 13.83 mg/dL DM CB: 178.08 ± 17.66 mg/dL non-DM CB: 86.56 ± 10.17 mg/dL	The study failed to prove the authenticity of GCB in assessment of patients with DM in dental chair using glucometer.
MV. Bhavsar	2016	India	70	60	DM GCB: 156.07 ± 49.23 mg/dL non-DM GCB: 90.8 ± 11.07 mg/dL DM CB: 156 ± 49.89 mg/dL non-DM CB: 93.41 ± 9.3 mg/dL	GCB may serve as a potential and excellent source for screening of blood glucose during routine periodontal examination in populations with known and unknown history of DM.
Sarita Parihar	2016	India	70	60	DM GCB: 156.07 ± 49.23 mg/dL non-DM GCB: 90.8 ± 11.07 mg/dL DM CB: 166.61 ± 52.18 mg/dL non-DM CB: 101.35 ± 13.05 mg/dL	GCB collected during diagnostic periodontal examination may be an excellent source of blood for glucometric analysis.
Rajesh	2016	India	24	45,8	Not reported	Capillary blood glucose level could be estimated using the regression equation: Capillary blood glucose = 84.66 + 0.77x gingival crevicular blood glucose level
M. D. Shylaja	2016	India	30	50	Fasting GCB: 110 ± 14 mg/dL Post prandial GCB: 163 ± 18 mg/dL Fasting CB: 109 ± 18 mg/dL Post prandial CB: 158 ± 20 mg/dL	GCB and CB showed positive correlation.
Siluvai Sibyl	2017	India	30	53,3	Not reported	GCB is one of the earliest sources for screening DM in dental office but not as an alternative to other measurements.
Biagio Rapone	2020	Italy	140	NA	DM GCB: 160.42 ± 31.31 mg/dL non-DM GCB: 93.93 ± 20.93 mg/dL DM CB: 161.64 ± 31.56 mg/dL non-DM CB: 90.88 ± 19.38 mg/dL	Testing GCB may be an advantageous tool in detecting patients with DM.
Abhijeet R. Sande	2020	India	100	31	Not reported	GCB can be an excellent source of blood for analysis of blood glucose levels.
Quratulain Saeed	2021	Pakistan	348	58	GCB: 151 ± 60.5 mg/dL CB: 159.8 ± 62 mg/dL	GCB and CB were moderately correlated, while HbA1c scores had a strong correlation with tooth mobility.
Juan Wu	2021	China	60	56,67	GCB: 7.96 ± 3.56 mmol/L; 6.03 ± 1.58% HbA1c CB: 8.09 ± 3.81 mmol/L; 6.01 ± 1.48% HbA1c	GCB can be used to estimate blood glucose and HbA1c level

**Abbreviations: DM**: Diabetes mellitus; GCB: gingival crevicular blood; CB: capillary blood

### Risk of bias and level of evidence in studies

**Fig. 3:**
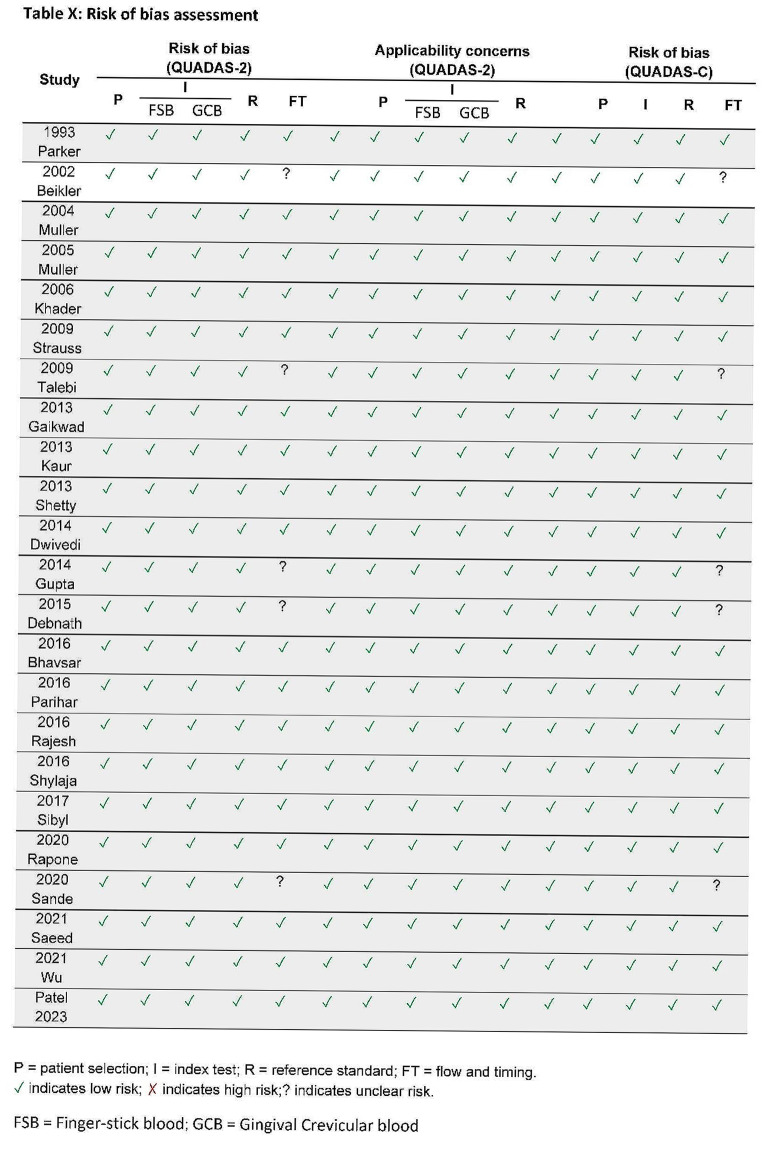
Risk of bias assessment of the included studies

Based on the QUADAS-C risk of bias tool, the included studies received low risk of bias, only five studies missed on reporting the time between index and reference test, although it did not affect overall risk of the studies (Fig. 3). According to the classification system described by Wright et al., we assigned level III evidence for all the included articles. Referring to this classification system under the diagnostic research category, studies of nonconsecutive patients (without consistently applying the reference gold standard) should be considered as level III evidence.

### Results of individual studies

Only one study has reported on HbA1c level measurements in patients with and without DM in severe and moderate periodontitis. The GCB and CB values in patients with DM were 7.72% ± 1.71%, and 7.89% ± 1.78%, while in patients without DM, the values were 5.28% ± 0.3%, and 5.23% ± 0.32%, respectively. There were highly significant correlations between the measurements with values of *r* = 0.977, and *r* = 0.829, respectively [14]. Due to the insufficient number of studies, performing quantitative analysis on HbA1c measurements was not possible.

18 studies have reported on the specific mean glucose levels measured from GCB and capillary blood (CB) with 12 different glucometers, the most frequently used glucometer was Accu-Check in 4 studies. 16 studies have reported their outcomes in mg/dl while two in mmol/l which were converted to mg/dl by the authors. The highest and lowest mean values recorded from GCB was 243.27 and 156.07 mg/dl in the DM group and 118.76 and 90.08 mg/dl in the non-DM group respectively [12, 25–35, 37, 40, 41, 43–45]. Two of the studies have found statistically significant differences between GCB and CB values (*p* = 0.001) [33, 37]. The first study’s mean values for the DM group were 210.56 ± 17.26 mg/dl 178.08 ± 17.66 mg/dl and for the non-DM group 118.76 ± 13.83 mg/dl 86.56 ± 10.17 mg/dl respectively [33]. The second study reported a mean value of 77.94 ± 38 mg/dl for GCB and 102.96 ± 37 mg/dl for CB [37].

One study has reported on the exact periodontal status of the patients as gingival indexes and probing depths. Patients with DM had higher gingival index and probing depth values compared to non-DM patients with 2.18 ± 0.39 and 4.43 ± 0.97 mm against 1.77 ± 0.28 and 3.96 ± 0.75 mm respectively [29].

### Results of synthesis

**Fig. 4:**
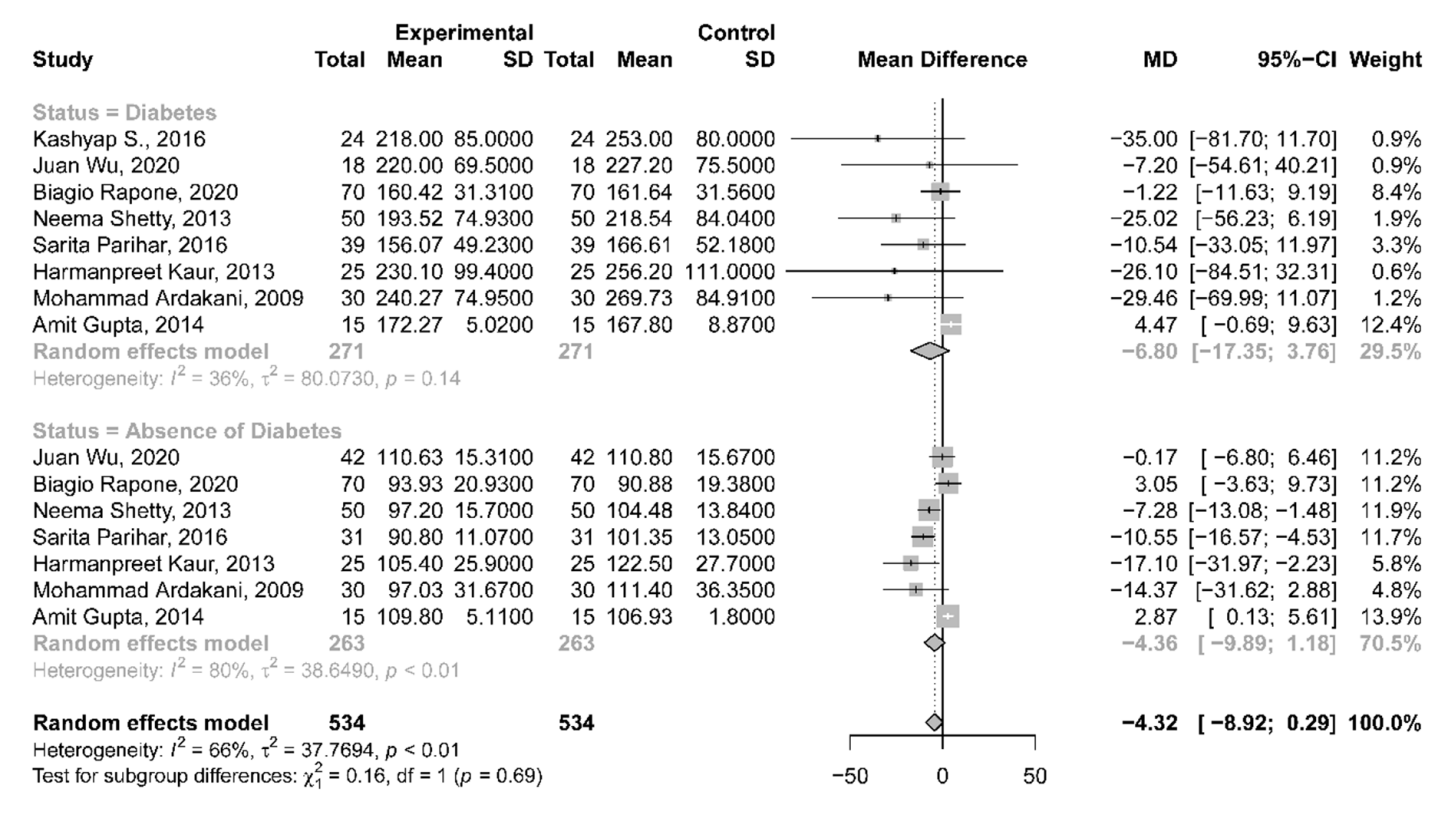
Forest plot representing the differences between the means of GCB and CB glucometer readings in patients with and without DM

Meta-analysis was performed to calculate the differences between the mean values of blood glucose level measurements between GCB and CB sampling sites in patients with and without DM. The analysis did not yield statistically significant differences with values of -6.80 [-17.35; 3.76] and − 4.36 [-9.89; 1.18] in patients with and without DM, respectively. The heterogeneity of the analysis remained low in the DM patient group with an I2 value of 36%, whereas high heterogeneity was detected in the non-DM patient group (I2 = 80%) (Fig. 4).

**Fig. 5:**
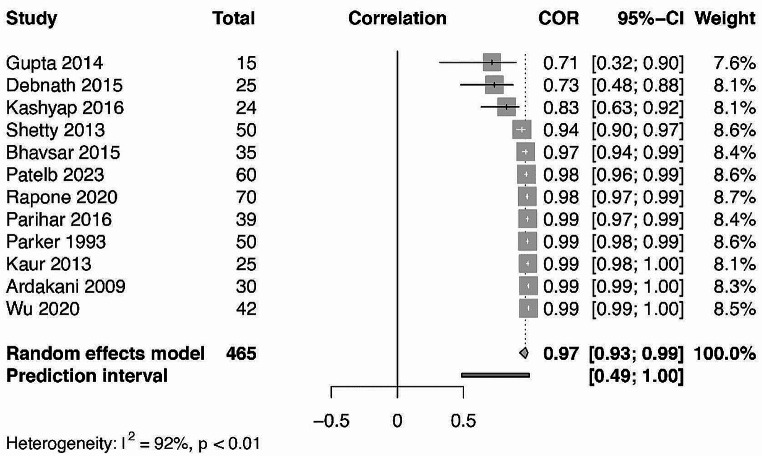
Forest plot representing the correlation between GCB and CB glucometer readings in patients with DM

**Fig. 6:**
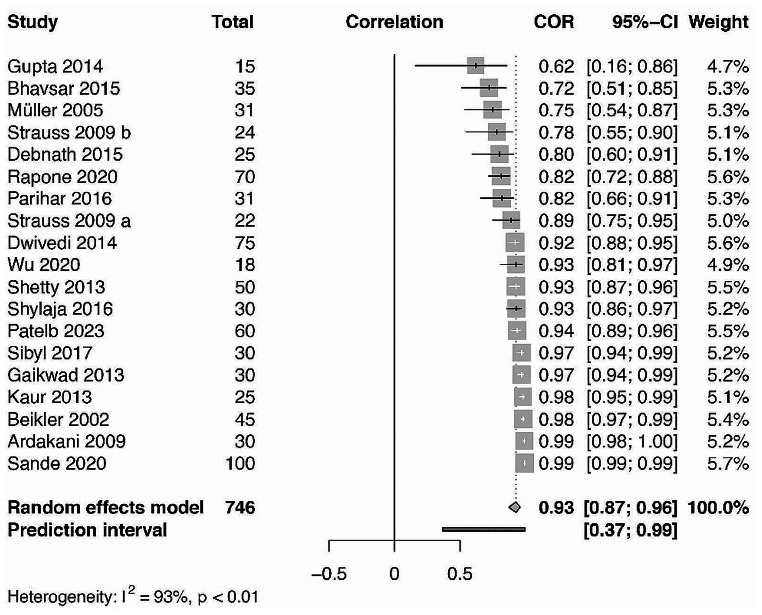
Forest plot representing the correlation between GCB and CB glucometer readings in patients without DM

Subsequently, quantitative analysis was performed on the Pearson’s correlations between GCB and CB glucose level values in DM and non-DM patients’ groups (Figs. 5 and 6.). 16 and 12 studies have reported on the necessary data for non-DM and DM patient group values respectively. Statistically significant (*p* = 0.001) correlations were found for the DM using the random effects model with a value of 0.97 [0.927; 0.987] using 95% confidence intervals with substantial heterogeneity I^2^ = 91,5%. Similar statistically significant (*p* = 0.001) results were found for the non-DM patient groups with a value of 0.927 [0.873; 0.958] with substantial heterogeneity I^2^ = 93.2%.

## Discussion

A systematic review and meta-analysis were performed on the reliability of GCB glucose and HbA1c measurement to assess glycemic control and identify patients with DM. The meta-analysis has found statistically significant correlations between GCB and CB in both DM and non-DM patient groups.

In order to assess a patient’s chronic glycemic control HbA1c is the easiest and most important value to measure, hence It quantifies the last two to three months of average blood glucose levels. However only assessing a patient’s glycemic control based on HbA1c can be misleading, since a given HbA1c value can be associated with wide ranges of mean glucose values, therefore knowing the patients current mean glucose level can help to interpret the meaning of actual HbA1c levels [46]. Only one study by Wu et al. have compared the HbA1c values from GCB to CB using an ion-exchange high-performance liquid chromatography where they have found highly significant correlations even with elevated HbA1c levels in patients with different degrees of periodontitis. In order to confirm the appropriateness of HbA1c level measurements from GCB additional studies are required [44].

Chair-side glucose meters are an increasingly popular option to assess average blood glucose levels in a time [47]. A study conducted by Ekhlaspour et al. in 2016 have compared the accuracy of 17 widely available glucometers to ISO standards and only 7 and 2 glucometers had the diagnostic accuracy to meet the ISO 2003 and 2013 standards respectively [48]. The U.S. Food and Drug Administration have updated the requirements for diagnostic accuracy in 2019 with requirements of 95% within +/- 15% across the measuring range and 99% within +/- 20% across the measuring range, therefore the use of glucometers with the updated FDA regulations are highly advised to gather correct glucose readings [49].

Most of the examined studies are supporting the use of GCB to measure blood glucose levels. The main advantages of using GCB as the source is the time-effectiveness that it can be done during routine periodontal examination by the dentist while not requiring to wait long times for the results [12]. Secondly, the cost-effectiveness of the procedure plays a major role in the wide-scale usability of this technique, since the purchase of glucometers and test-strips are very modest [39]. Thirdly the patient’s comfort is an important aspect of the procedure hence there is no need for an additional finger puncture therefore blood glucose measurements can be done without any pain or inconvenience [42]. Even though our analysis has found significant correlations between the results of the different sampling sites, we also found differences between the means that are not statistically significant, but in certain cases can prove to be clinically relevant. These differences are larger in patients with DM compared to patients without DM. Therefore, this procedure is unsuited to accurately assess glycemic control in patients with DM, however it may be useful to screen undiagnosed cases. According to the ADA, FBG is below 130 mg/dL in patients without DM, and the majority of patients have values between 70 and 100 mg/dL. Hence, GCB can possibly be useful to detect blood glucose levels in the DM range in the majority of the population despite the differences. Although, it is important to note, when high glucose levels are present, the readings should be confirmed by conventional CB measurements as well. Unfortunately, the prandial status significantly affects the patient’s glycemic levels at the time of measurement, and it proves to be a significant limitation of this procedure [50]. To overcome this, measuring HbA1c level would be more prominent, since it is unaffected by the patient’s prandial status, and can provide a more accurate view on the patient’s overall systemic glycemic load of the last two to three months [51]. Due to the very limited amount of evidence on HbA1c level measurements from GCB, it is not possible to draw any definitive conclusions, but the results are promising, and more prospectively designed studies are necessary to allow for quantitative analysis. One of the most important limitations is that gingival inflammation is necessary to be present in order to obtain sufficient amount of GCB for the test strip after the periodontal examination, since elevated bleeding is usually present with advanced inflammation. Most available glucometers need at least 4 µL blood to give correct readings [12]. Three studies concluded that GCB cannot be used as a source to measure blood glucose levels. Müller et al. have received error readings in every 3rd case due to the low amounts of GCB and found significant differences between GCB and CB measurements. These differences may have been caused by the low GCB volumes diluted by gingival fluids [36, 37]. The study conducted by Debnath et al. has found statistically significant differences between GCB and CB and found very low correlation values. In that study GCB readings were consistently higher compared to CB readings, which may have been because of lower amounts of gingival blood and higher amounts of gingival fluids in the source of measurement. The lower amounts of GCB could have been present because of the inclusion of patients with very mild cases of periodontitis [33].

There have been several outliers in our analysis on the correlation between GCB and CB readings which have caused high heterogeneity in the analysis. Low correlations between readings could be explained in several studies by the low amounts of GCB and possible contaminations by gingival fluid [33, 35, 36]. In the study conducted by Bhavsar et al. lower correlation values were found only in the non-DM patient group, which could be explained by the significantly lower GI in the non-DM groups, therefore GCB amounts may have been lower in the non-DM group [29]. Strauss et al. have compared the correlations of GCB and CB measurements and found significant differences between patients with milder and more severe periodontitis. Patient groups with smaller probing depths have presented lower correlation values, the cause for this discrepancy could be answered with the unsatisfying amount of GCB which may result in imprecise readings [39].

### Strengths and limitations

The strength of our study is that we could include high number of studies for both quantitative and qualitative analysis and it is the first systematic review on this topic to the best of our knowledge. Our limitation is that in our analysis the heterogeneity remained very high which could be explained by the different glucometers used, different types and severity of DM and periodontitis in the patient groups. Unfortunately, this issue prevented us from presenting more thorough analyses of the blood glucose and HbA1c values reported by the included studies as well as some further analysis, including comparing concordance, or measurement repeatability. The last but not least limitation of our study is the level of evidence in the included studies (level III) that warrants cautious conclusions.

### Implications for future research

In order to increase the certainty of evidence provided by clinical studies on the topic studies with more rigorous methodologies are required. The type of glucometers used and the severity of periodontal inflammation heavily influences the accuracy of readings, therefore the use of FDA approved glucometers and similar periodontal status matched cohorts are highly suggested for future studies. More studies are required to assess the accuracy of GCB readings regarding HbA1c levels. HbA1c gives an overview of patient blood glucose levels of the last two to three months, therefor it is widely used of DM diagnosis.

## Conclusion

We have found that gingival crevicular blood could be used to measure blood glucose levels to identify patients with undiagnosed diabetes, if the necessary amount of uncontaminated gingival blood is present for a correct reading. However, the procedure is unsuited to monitor glycemic control in patients with diabetes, due to the higher inaccuracies in elevated glucose levels. When a glucose level in the hyperglycemia range (> 130 mg/dL) is detected in a patient without diabetes, conventional finger prick blood measurement is advised for validation. It is important to emphasize the use of FDA approved glucometers in order to ensure the accuracy of all measurements.

### Clinical relevance

#### Scientific rationale for study

Diabetes mellitus is a well-known risk factor of developing severe periodontitis. More than half of the patients living with diabetes remain undiagnosed. The link between periodontitis and diabetes makes periodontal screening a perfect opportunity to identify patients with underlying diabetes. There is no previous comprehensive review of the available literature on the topic of investigating the reliability of gingival crevicular blood on blood glucose and HbA1c level measurements.

#### Principal findings

We have found statistically significant correlations between gingival crevicular blood and capillary blood glucose measurements, and statistically insignificant, but clinically relevant differences between the mean values.

#### Practical implications

When a necessary amount of gingival blood is present, an FDA approved glucometer can be used to screen for elevated blood glucose levels in patients without diabetes. In case of post prandial measurement, higher than 140 mg/dL value suggests that hyperglycemia is present, and conventional finger prick testing is necessary to verify the results. It is important to note, that due to the low quality of the studies, the certainty of evidence remains low.

## Data Availability

The datasets used in this study can be found in the full-text articles included in the systematic review and meta-analysis.

## Declarations

### Statement of clinical relevance

• From more than 500 million patients with diabetes mellitus 50% are undiagnosed.

• Diabetes mellitus is a significant risk factor of periodontitis.

• Using Gingival crevicular blood provided on routine periodontal probing may prove to be a great opportunity to identify underlying diabetes mellitus.

• This method could be less unpleasant, alternative with less pain and discomfort for the patient.
